# Genetic screening for hereditary transthyretin amyloidosis with polyneuropathy in western Sicily: Two years of experience in a neurological clinic

**DOI:** 10.1111/ene.16065

**Published:** 2023-09-19

**Authors:** Vincenzo Di Stefano, Antonino Lupica, Paolo Alonge, Antonia Pignolo, Sofia Maria Augello, Francesca Gentile, Andrea Gagliardo, Francesca Giglia, Daniele Brinch, Maria Cappello, Daniela Di Lisi, Giuseppina Novo, Eugenia Borgione, Carmela Scuderi, Filippo Brighina

**Affiliations:** ^1^ Section of Neurology, Department of Biomedicine Neuroscience, and Advanced Diagnostics, University of Palermo Palermo Italy; ^2^ Unit of Neurology With Stroke Unit S. Giovanni di Dio Hospital Agrigento Italy; ^3^ Section of Gastroenterology and Hepatology, Department of Health Promotion, Mother and Child Care, Internal Medicine, and Medical Specialties University of Palermo Palermo Italy; ^4^ Section of Cardiology, Department of Health Promotion, Mother and Child Care Internal Medicine, and Medical Specialties, University of Palermo Palermo Italy; ^5^ Unit of Neuromuscular Diseases Oasi Research Institute, IRCCS Troina Italy

**Keywords:** amyloid, genetics, neuromuscular, neuropathy, neurophysiology

## Abstract

**Background and purpose:**

Hereditary transthyretin amyloidosis with polyneuropathy (ATTRv‐PN) is caused by mutations in the *TTR* gene, leading to misfolded monomers that aggregate generating amyloid fibrils.

**Methods:**

A prospective systematic genetic screening for ATTRv‐PN was proposed in patients presenting with a sensory–motor idiopathic polyneuropathy and two or more “red flags” among the following: family history of polyneuropathy or cardiopathy, bilateral carpal tunnel syndrome, cardiac insufficiency, renal amyloidosis, lumbar tract stenosis, autonomic dysfunction, idiopathic gastrointestinal disease, amyloid deposits on biopsy, and vitreous opacities. The detection rate was calculated, and nonparametric analyses were carried out to underline differences among screened positive versus negative patients.

**Results:**

In the first step, 145 suspected patients underwent genetic testing, revealing a diagnosis of ATTRv‐PN in 14 patients (10%). Then, cascade screening allowed early recognition of 33 additional individuals (seven symptomatic ATTRv‐PN patients and 26 presymptomatic carriers) among 84 first‐degree relatives. Patients with a positive genetic test presented a higher frequency of unexplained weight loss, gastrointestinal symptoms, and family history of cardiopathy.

**Conclusions:**

A systematic screening for ATTRv‐PN yielded an increased recognition of the disease in our neurological clinic. Unexplained weight loss associated with axonal polyneuropathy had the highest predictive value in the guidance of clinical suspicion. A focused approach for the screening of ATTRv‐PN could lead to an earlier diagnosis and identification of asymptomatic carriers, who will be promptly treated after a strict follow‐up at the clinical onset.

## INTRODUCTION

Hereditary transthyretin amyloidosis with polyneuropathy (ATTRv‐PN) is an adult onset, rare, and multisystemic disease, affecting the sensorimotor and autonomic functions along with other organs, especially the heart, gastrointestinal tract, eye, and kidney [1, 2]. ATTRv‐PN is caused by the accumulation of abnormal amyloid fibrils originating from mutations in the *TTR* gene; *TTR* mutations display incomplete penetrance and present an autosomal dominant pattern of inheritance [3, 4]. Studies on the Val30Met variant have shown mean interval from disease onset to death to be 7.3 years, with cardiomyopathy being the major cause of death in the absence of treatment [2]. Clinical phenotype is heterogeneous and often unpredictable; therefore, the diagnosis is very difficult and, in most cases, delayed. Consequently, misdiagnosis of ATTRv‐PN carries high costs for the community in terms of mortality and inappropriate treatments [5], although several available treatment options are particularly effective in treating early disease stages [6]. In the recent past, the diagnosis of ATTRv‐PN required genetic testing performed in the presence of a strong clinical suspicion and a positive biopsy [5, 7]. However, the role of biopsy has become questioned due to the broad availability of genetic testing [8, 9]. More recent diagnostic algorithms suggest anticipating and often replacing the biopsy in the diagnostic workup [1]. Consequently, based on published literature and expert opinions, symptom clusters and specific “red flags” have recently been proposed to facilitate an earlier diagnosis [10, 11]. However, there is still a need for new strategies to find undiagnosed individuals and implement existing evidence‐based guidelines to improve ATTRv‐PN care. Recent evidence suggests that ATTRv‐PN should be suspected if progressive peripheral sensory–motor neuropathy is observed in combination with one or more of the following conditions: autonomic dysfunction (erectile dysfunction, orthostatic hypotension, syncope), cardiomyopathy, gastrointestinal symptoms, unexplained weight loss, bilateral carpal tunnel syndrome, lumbar canal stenosis, renal impairment, ocular involvement (vitreous opacities), and/or family history of polyneuropathy, cardiomyopathy, or ATTRv‐PN [1, 10]. Finally, because ATTRv‐PN is an autosomal dominant genetic condition, screening at‐risk relatives of individuals with ATTRv‐PN (*cascade* testing) is highly effective in identifying additional individuals who require treatment [11]. In this study, we performed genetic screening on patients presenting to our clinic for neuromuscular diseases with a sensory or sensory–motor polyneuropathy and one or more clinical features suggesting ATTRv‐PN. We hypothesized that a systematic screening for ATTRv‐PN might contribute to significantly reducing the diagnostic delay of ATTRv‐PN in nonendemic areas, as well as ensuring early treatment for this rare inherited disease.

## METHODS

### Study procedures

The study was approved by the Ethical Committee of Palermo (Sicily region, Italy) on 13 July 2020 (V. n.7/2020) and was conducted in conformity with the Declaration of Helsinki principles; all participants gave their written informed consent to participate in the study.

This prospective study aimed to evaluate the role of genetic testing in the diagnosis of ATTRv‐PN at a centre specializing in neuromuscular diseases to establish the impact of ATTRv‐PN in a real‐life context. Patients suspected to have ATTRv‐PN based on specific “red flags” [10, 11] were enrolled over a 2‐year period. In a second phase, they went through genetic testing. Clinical data were collected from patients undergoing transthyretin genotyping in a neurological clinic specializing in the diagnosis and care of neuromuscular diseases in Palermo (Policlinico Paolo Giaccone). For each patient undergoing genetic testing, the presence of specific “red flags” was investigated through a detailed questionnaire (see Online Resource 1: Genetic screening questionnaire for hereditary transthyretin amyloidosis with polyneuropathy [ATTRv‐PN]).

In screened positive patients, main misdiagnoses and disease duration were investigated and a cascade screening was proposed to first‐degree family members according to the predicted age of disease onset (PADO) (i.e., according with expected clinical onset) [12]. Then, clinical data were compared in patients with positive and negative genetics to identify the role of each “red flag” in a real‐life context.

### Patient population

Inclusion criteria were (i) informed consent for genetic testing; (ii) age > 18 years; and (iii) presence of polyneuropathy confirmed clinically and electrophysiologically and at least one “red flag” among the following: (a) bilateral carpal tunnel syndrome, (b) sensory ataxia, (c) lumbar canal stenosis, (d) idiopathic gastrointestinal disease, (e) unexplained weight loss, (f) autonomic dysfunction (erectile dysfunction, orthostatic hypotension, syncope), (g) cardiomyopathy, (h) renal impairment, (i) ocular involvement (vitreous opacities), and/or (j) family history of polyneuropathy, (k) cardiomyopathy, or (l) ATTRv‐PN. Exclusion criteria were (i) lack of informed consent, (ii) identification of other cause for neuropathy, and (iii) ineligibility for genetic testing. For cascade screening, we proposed a molecular genetic test according to expert consensus statements and updated guidelines, considering the PADO (i.e., we screened patients with P64L after 55 years according to expected clinical onset) [1, 5, 10].

### Statistical analysis

The statistical analysis was applied to compare the *TTR* mutation groups (negative and positive). In particular, the Mann–Whitney *U*‐test was used to test the only continuous variable (age), and interquartile range was reported for the overall population, and negative and positive groups. The chi‐squared test was applied for the other categorical variables, and the proportions were reported also for the overall population, and negative and positive groups. A comparison between screened positive and negative patients was performed for each of the “red flags” and clinical features. For statistical analysis, a *p*‐value < 0.05 was considered significant.

## RESULTS

**FIGURE 1:**
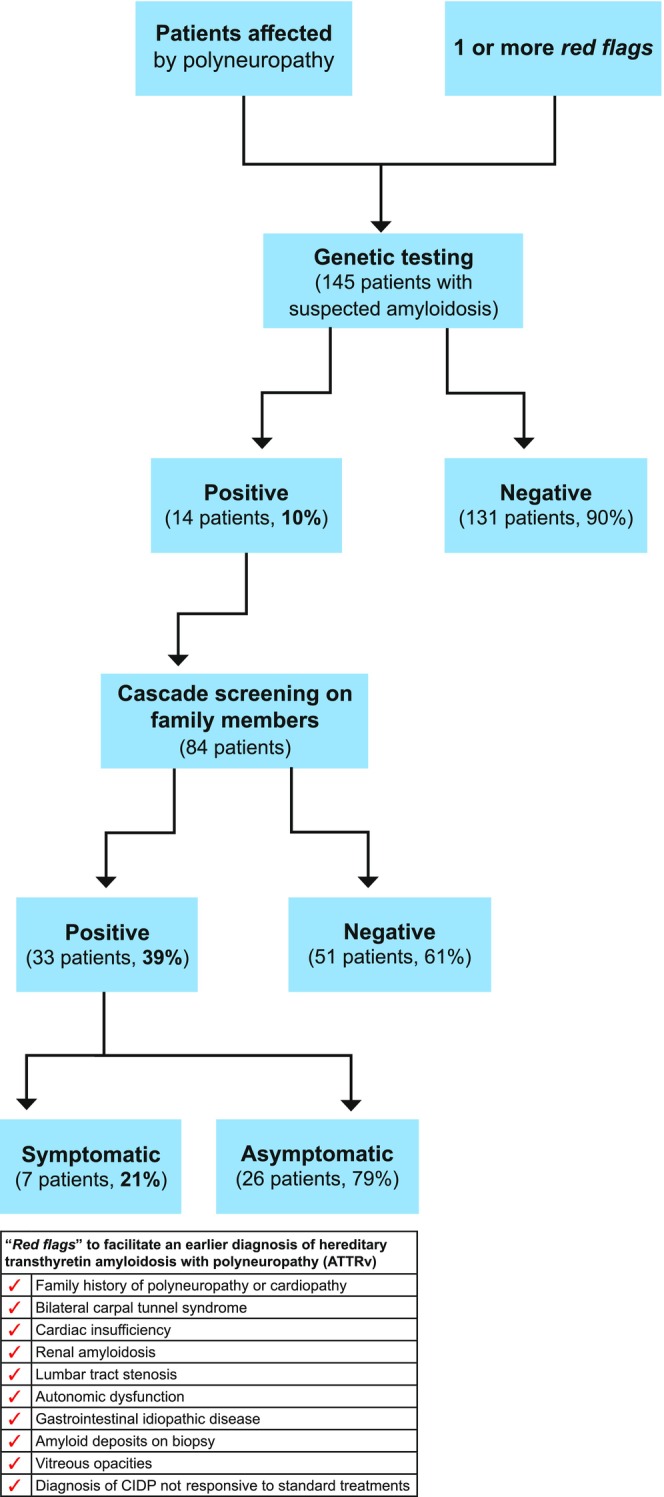
Screening procedures in patients affected by polyneuropathy. CIDP, chronic inflammatory demyelinating polyradiculoneuropathy.

The genetic screening performed in the presence of clinical symptoms of ATTRv‐PN allowed a diagnosis of ATTRv‐PN in 14 families and early recognition of 21 symptomatic ATTRv‐PN patients and 26 asymptomatic carriers of the *TTR* mutation. Figure 1 reports screening procedures and enrolled patients.

### Phase 1: Screening patients affected by polyneuropathy

**FIGURE 2:**
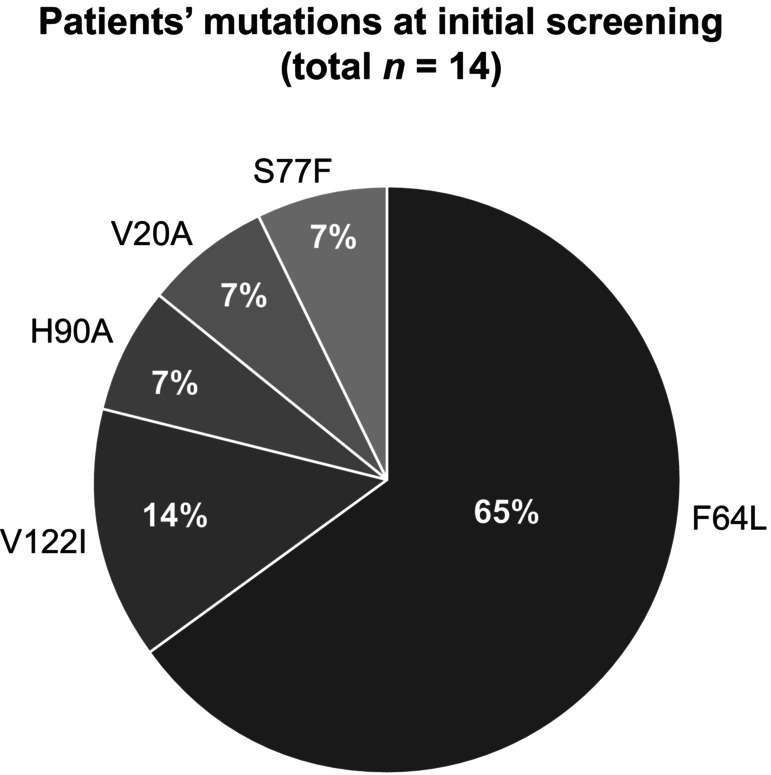
Principal mutations encountered in the study sample.

Because of systematic screening, 145 patients affected by neuropathy underwent genetic testing, which were positive for mutations in the *TTR* gene in 14 cases (10%). Five mutations occurred among ATTRv‐PN patients, with Phe64Leu being the most frequent genotype (Figure 2).

Interestingly, His90Asn, Val20Ala, and Ser77Phe were reported for the first time in Sicily. Table 1 compares clinical findings in patients with positive and negative results on genetic testing. *TTR*‐mutated patients presented a higher frequency of unexplained weight loss (*p* = 0.007), gastrointestinal symptoms (*p* = 0.05), and family history of cardiomyopathy (*p* = 0.05).

**TABLE 1:** Clinical features in screened positive versus negative patients.

Clinical features	ATTRv patients	Patients with negative genetic testing	*p*
Age, years	71.6 ± 7.3	64.5 ± 12.1	0.041*
Gender, males	8 (57%)	81 (62%)	0.47
Unexplained weight loss	9 (64%)	36 (27%)	0.007*
Bilateral carpal tunnel syndrome	10 (71%)	62 (47%)	0.07
Ataxia	8 (57%)	65 (50%)	0.4
Lumbar canal stenosis	3 (21%)	31 (24%)	0.57
Gastrointestinal disturbances	9 (64%)	50 (38%)	0.05*
Autonomic dysfunction	9 (64%)	65 (50%)	0.22
Cardiopathy	5 (35%)	39 (30%)	0.42
Renal dysfunction	1 (7%)	17 (13%)	0.44
Ocular disorders	3 (21%)	35 (27%)	0.47
Family history of neuropathy	3 (21%)	16 (12%)	0.2
Family history of cardiopathy	8 (57%)	41 (31%)	0.05*

Abbreviation: ATTRv, hereditary transthyretin amyloidosis. *p* < 0.05

Patients carrying *TTR* mutations were older (Table 1). Also, bilateral carpal tunnel syndrome (CTS; 10 patients, 71%), associated with unexplained weight loss, gastrointestinal disturbances, and autonomic dysfunction (nine patients, 64%), were the most recurring “red flags” among *TTR*‐mutated patients. Of note, ataxia and family history of cardiomyopathy were present in 64% and 57% of positive cases, whereas family history of neuropathy was uncommon (21%). Renal and ocular dysfunction, and spinal lumbar stenosis, were reported in a minority of cases. Finally, patients screening positive on genetic testing presented less frequently with diabetes and autoimmune comorbidities compared to negative ones. Anorexia was the most common misdiagnosis (7%), followed by motoneuron disease (5%). Table 2 shows misdiagnoses in ATTRv‐PN patients detected through systematic screening.

**TABLE 2:** Misdiagnoses in hereditary transthyretin amyloidosis patients, *N* = 14.

Misdiagnosis	*n* (%)
Anorexia nervosa	4 (29)
ALS	3 (21)
CIDP	2 (14)
Idiopathic polyneuropathy	2(14)
Diabetes	1 (7)
Cardiomyopathy	1 (7)
Lumbar canal stenosis	1(7)

Abbreviations: ALS, amyotrophic lateral sclerosis; CIDP, chronic inflammatory demyelinating polyradiculoneuropathy.

### Phase 2: Cascade screening on first‐degree relatives with ATTRv‐PN

Through the diagnosis of 14 ATTRv‐PN symptomatic patients, 14 families were detected. After diagnosis of the probands, genetic screening was proposed to their first‐degree relatives. Genetic testing was proposed to 104 relatives, but only 84 subjects (80%) gave consent for genetic testing. As a result, 33 individuals carrying a *TTR* mutation were detected (39%). After clinical and neurophysiological evaluation, seven patients (21%) had evidence of sensory–motor polyneuropathy and were started on treatment, whereas 26 presymptomatic carriers of *TTR* mutation began regular follow‐up.

## DISCUSSION

**FIGURE 3:**
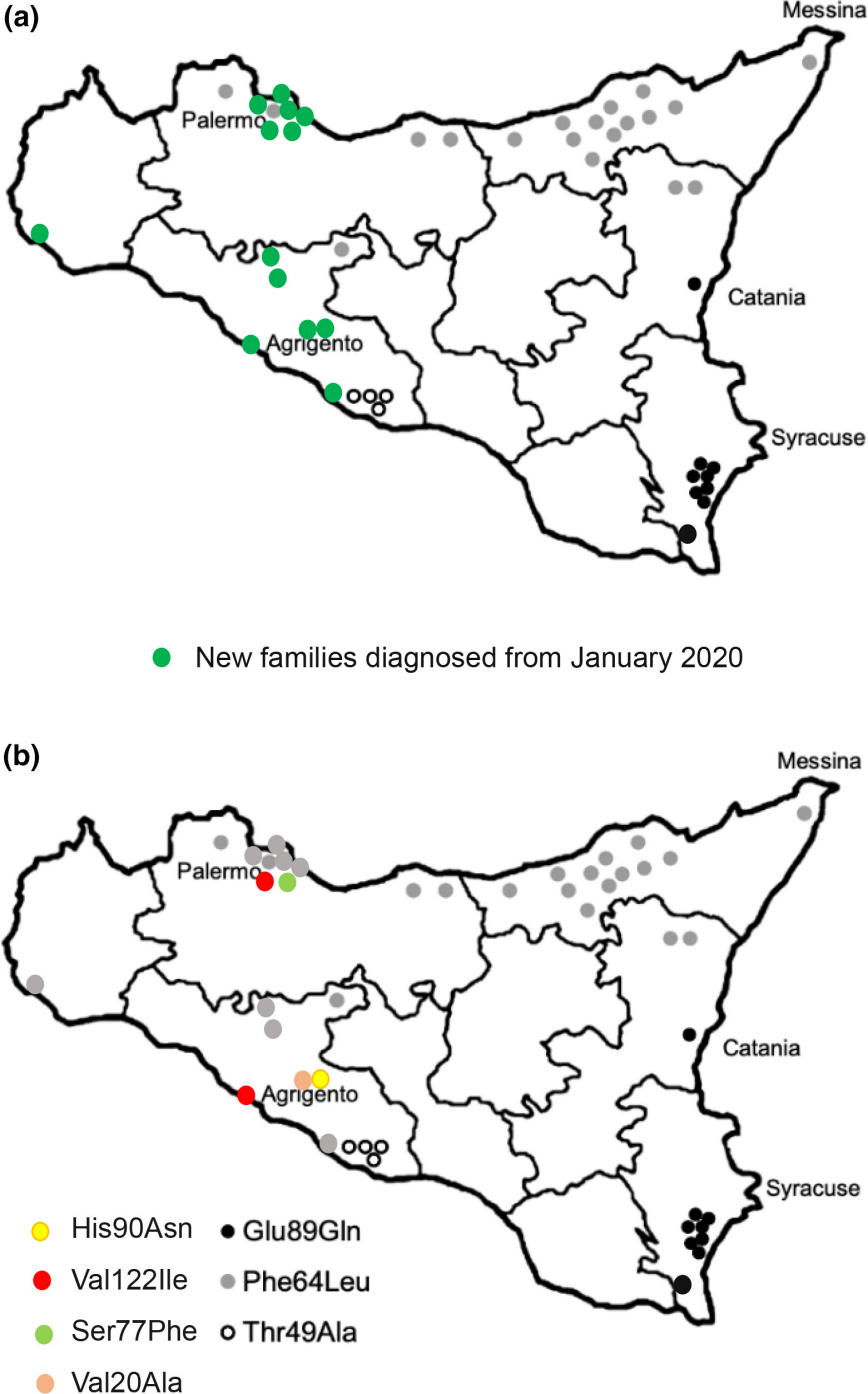
Geographical distribution of *TTR* mutations in Sicily. Modified from Gendre and Planté‐Bordeneuve [15].

This study systematically evaluated the impact of clinical “red flags,” described in pivotal papers and guidelines, in predicting ATTRv‐PN diagnosis in a real‐life setting. Genetic screening was offered to patients presenting with a sensory or sensory–motor polyneuropathy and one or more clinical features suggesting ATTRv‐PN. The main result that should be underlined is that ATTRv‐PN is not uncommon in Sicily; a detection rate of 10% should encourage the clinical suspicion of such a treatable disease, especially considering its high mortality rates and costs for society. As expected from the data of the Italian registry [13], most ATTRv‐PN patients carried a Phe64Leu mutation (64%) and two subjects a Val122Ile mutation (14%). However, comparing the distribution of mutations found in a previous study [14], several differences emerge; in our study, Phe64Leu frequency is almost twice as common, whereas the most prevalent mutation reported in that study (Glu98Gln) is absent in our population [14]. Furthermore, we describe four mutations that were previously unreported in Sicily (Figure 2). Among them, Ser77Phe has already been clearly reported as pathogenetic [15, 16, 17], whereas for Val20Ala a single report supported a pathogenetic role [18] and high confusion exists for the His90Asn variant [19, 20, 21, 22, 23]. Conversely, the pathogenic role of the His90Asn variant in our patient was confirmed by the presence of amyloid deposits in a salivary gland biopsy and a good response to RNA silencers. The presence of a different genetic background in the population referring to our clinic might explain these results, as they are descendants from western Sicilian ancestry (Palermo, Agrigento, and Trapani), whereas the previous Sicilian study mostly examined people from northern and eastern Sicily (Messina, Siracusa, and Catania) [14]. These new findings add new insights on the geographical distribution of ATTRv‐PN in our region (Figure 3).

**FIGURE 4:**
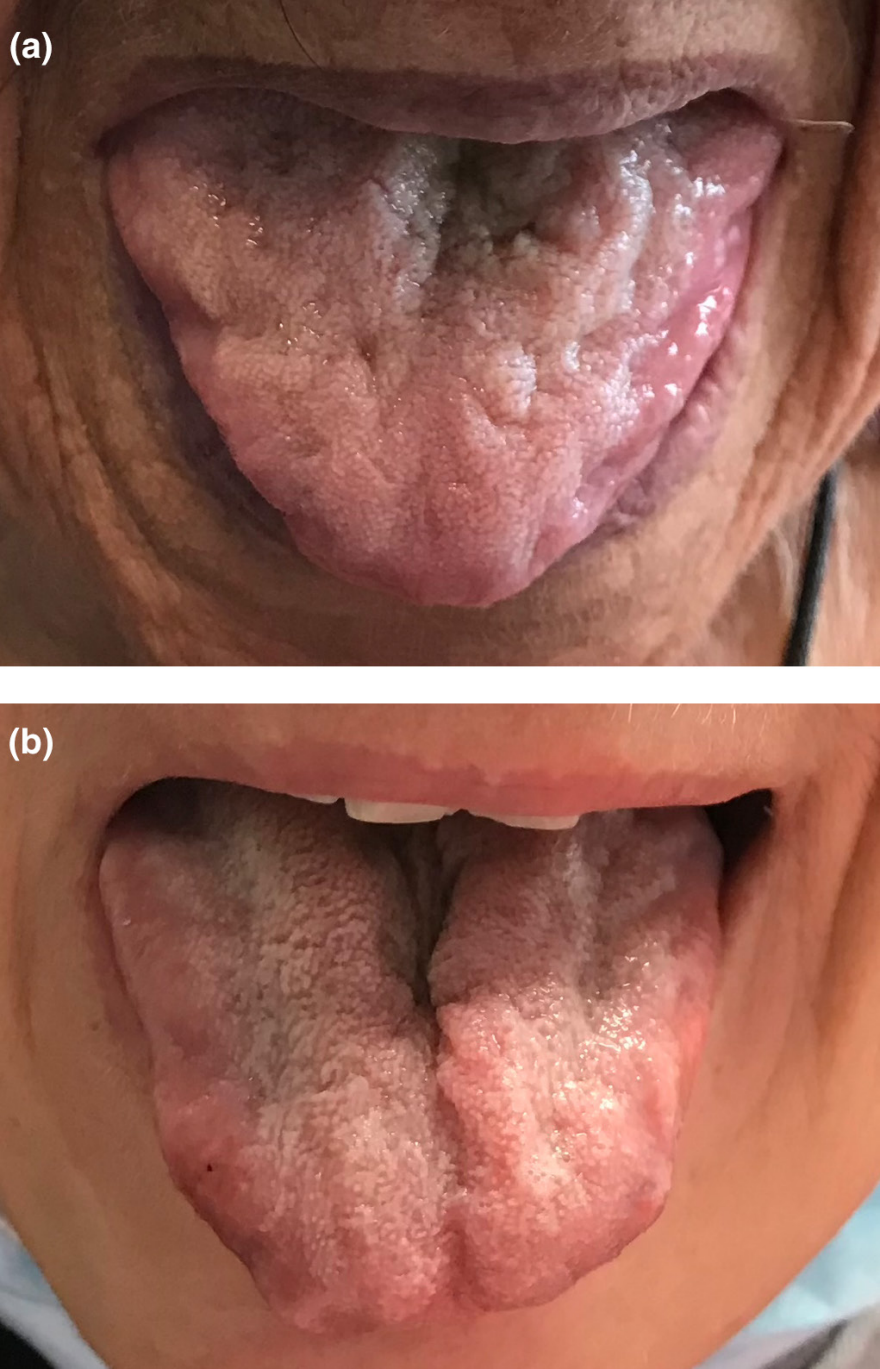
Tongue atrophy in two patients carrying Phe64Leu mutation in the *TTR* gene misdiagnosed with amyotrophic lateral sclerosis. (a) A woman with a history of depression, anxiety, and weight loss of 10 kg in 1 year complaining of difficulty in ambulation and dysphagia. (b) A woman with motor neuropathy and dysphagia.

From a diagnostic perspective, the clinical focus should be on misdiagnosis of ATTRv‐PN. Unfortunately, in such cases, the clinical presentation makes it difficult to distinguish ATTRv‐PN from other conditions, thus causing significant misdiagnosis [24]. Chronic inflammatory demyelinating polyradiculoneuropathy (CIDP), diabetes, sensory ataxia, and amyotrophic lateral sclerosis (ALS) are commonly considered misdiagnoses of ATTRv‐PN [24]. Conversely, the most frequent misdiagnoses in our study were anorexia nervosa (4/14) and ALS (3/14); other misdiagnoses were CIDP (2/14), idiopathic neuropathy (2/14), diabetes, cardiomyopathy, and lumbar canal stenosis (1/14 each). It should be noted that CIDP was not confirmed as the most common misdiagnosis in our study, whereas anorexia and ALS explained 50% of cases. These results might be explained by the presence of axonal motor neuropathy and high frequency of weight loss and gastrointestinal symptoms in our population. Of note, in two cases, severe tongue atrophy (Figure 4) and dysarthria were reported in two unrelated women carrying Phe64Leu mutations and motor neuropathy.

In such difficult cases, mild sensory deficits associated with depression have been overlooked due to the accompanying picture of relevant weight loss and motor impairment. Gastrointestinal involvement is frequent in ATTRv‐PN [25], and diarrhea, constipation, or weight loss may be present from the onset of the disease, even anticipating neurological symptoms [26]. Furthermore, gastrointestinal symptoms are insidious and can be misinterpreted as common conditions such as irritable bowel syndrome or functional dyspepsia, thus causing a relevant diagnostic delay. Of interest, whereas irritable bowel syndrome, unexplained malabsorption syndrome, protein‐losing enteropathy secondary to ischemia, and celiac disease have been hypothesized to be among the most common gastrointestinal misdiagnoses [10], anorexia nervosa was reported only anecdotally mimicking amyloidosis [27] (Table 2), but was recently reported as highly predictive for ATTRv‐PN in a recent study employing machine learning [28]. Also, weight loss and gastrointestinal symptoms showed the same prevalence in *TTR*‐mutated patients (64%), confirming their possible pathophysiological correlation [29, 30]. However, the prevalence of gastrointestinal symptoms did not reach a significant difference between the two groups, although a trend emerged; further analyses on larger populations might confirm the relevance of gastrointestinal involvement in ATTRv‐PN. Bilateral CTS, despite being the most frequently recurring “red flag,” showed a high prevalence in both screened positive and negative patients, as it is also commonly associated with other causes of peripheral nerve disease (i.e., hereditary neuropathy, rheumatoid arthritis, diabetes mellitus, and hypothyroidism) [31, 32]. However, our data confirm its strong association with ATTRv‐PN and, thus, its relevance as a “red flag.” Similarly, autonomic dysfunction and ataxia were often reported, but are also common in other forms of polyneuropathies that show a specific association with amyloidosis [2]. Of interest, cardiomyopathy was frequently reported in the family history of *TTR*‐mutated patients (57%) but was uncommon in patients themselves (37%). This apparent inconsistency might be explained by considering that the cardiological phenotype can follow the onset of neuropathy in mixed phenotype patients, being hidden for several years [7]. The Phe64Leu genotype, which is characterized by predominant neurological involvement associated with mild cardiac involvement in advanced disease [14], was the most prevalent mutation in our study sample; therefore, we hypothesize that this phenotype might be more frequently found in older and unrecognized affected family members rather than in younger patients with a more recent onset of symptoms. Furthermore, cardiac uptake on bone scintigraphy resulted in only one patient diagnosis in this study. Musumeci et al. have linked Phe64Leu mutation to a low bone scintigraphy sensitivity for the diagnosis of amyloid cardiomyopathy, and our study seems to confirm this finding [33].

Finally, sex distribution deserves some consideration; although ATTRv‐PN is considered a disease prevalent in males [34], male to female (M:F) ratio was 6:8 in this sample of ATTRv‐PN patients. The Phe64Leu mutation was reported as predominant in females (M:F ratio = 1:22) in a population from Lazio [35]; in Sicily, a M:F ratio of 16:12 was reported [14]. It should be noted that the cited reports included asymptomatic carriers; similarly, our study included 26 asymptomatic carriers.

A last consideration from these data is the importance of cascade screening. Cascade screening of first‐degree relatives allowed prompt recognition of a further seven ATTRv‐PN patients who presented with mild sensory neuropathy that was undetected before and overlooked by clinicians. These “lucky” patients had the opportunity to be early treated in the first stage of the disease (familial amyloid polyneuropathy 1) with good response to treatment with an RNA silencer. Also, 26 asymptomatic carriers attend to regular follow‐up. It is reasonable to state that the prognosis of both symptomatic and asymptomatic carriers of *TTR* mutations will improve thanks to early diagnosis because of the availability of effective treatments.

## LIMITATIONS

Our study presents several limitations that should be addressed. The study sample size was quite small. These data refer to a screening period in a limited area of western Sicily in a tertiary referral neurological clinic (i.e., it is a clinic dealing with several rare neuromuscular disorders, not a common neurological clinic); hence, a selection bias was expected. Also, all patients came to our attention for neuropathy and were evaluated by a neurologist as a first approach; therefore, cardiac history will be either over‐ or underestimated. A further limitation comes from the concept of a “red flag,” which can be self‐reported by the patient, described in a specialist's report, or demonstrated in an instrumental examination with different grades of precision in the clinical assessment. Hence, the assessment of such “red flags” might be poor and incomplete (i.e., erectile dysfunction), due to underreporting or undervaluation. Finally, the lack of histopathological data might be a further limitation due to the risk of overdiagnosis of ATTRv‐PN in patients carrying variants with low penetrance. Patients positive for genetic testing might have polyneuropathy resulting from diseases other than amyloidosis. Further studies are needed to better define phenotype–genotype correlations in variants with low penetrance.

## CONCLUSIONS

ATTRv‐PN is a treatable inherited disease with late onset in nonendemic countries. Diagnosis of ATTRv‐PN is difficult, with a relevant diagnostic delay, misdiagnosis, and high costs for the community, but a systematic screening for ATTRv‐PN in the neurological setting might contribute to significantly reducing the diagnostic delay of ATTRv‐PN. Unexplained weight loss associated with gastrointestinal disturbances, family history of cardiomyopathy, and polyneuropathy represent the most common presentation of ATTRv‐PN in Western Sicily, whereas anorexia and ALS might represent overlooked misdiagnoses.

## AUTHOR CONTRIBUTIONS

**Vincenzo Di Stefano**: Conceptualization; Data curation; Writing—original draft; Methodology; Supervision; Validation; Writing—review & editing. **Antonino Lupica**: Validation. **Paolo Alonge**: Validation. **Antonia Pignolo**: Validation. **Sofia Maria Augello**: Validation. **Francesca Gentile**: Validation. **Andrea Gagliardo**: Validation. **Francesca Giglia**: Validation. **Daniele Brinch**: Validation. **Maria Cappello**: Validation. **Daniela Di Lisi**: Validation. **Giuseppina Novo**: Validation. **Eugenia Borgione**: Validation. **Carmela Scuderi**: Validation. **Filippo Brighina**: Conceptualization; Validation.

## FUNDING INFORMATION

This research was funded by the European Union—FESR or FSE, PON Research and Innovation 2014–2020—DM 1062/2021. Financial unconditional support for article processing fees and editorial assistance was provided by Alnylam Pharmaceuticals (Cambridge, MA, USA).

## CONFLICT OF INTEREST STATEMENT

None declared.

## Supporting information


DATA S1

## Data Availability

The data underlying this article will be shared on reasonable request to the corresponding author.
