# Supplementary material for: Genetic screening for hereditary transthyretin amyloidosis with polyneuropathy in western Sicily: Two years of experience in a neurological clinic
Source: Eur J Neurol. 2023 Sep 19;31(1):e16065. doi: 10.1111/ene.16065 (PMC11235612; doi:10.1111/ene.16065)
Supplement: Supplementary file 1 — DATA S1 [file ENE-31-e16065-s001.docx]

**Genetic Screening Questionnaire for Hereditary transthyretin amyloidosis with polyneuropathy (ATTRv-PN)**

Hereditary transthyretin amyloidosis with polyneuropathy (ATTRv-PN)

Sex ________

Age_________

**MISDIAGNOSIS SECTION**

- Do you suffer from autoimmune diseases? (e.g. Hashimoto thyroiditis, type I diabetes, celiac disease, rheumatoid arthritis, etc)

- YES
- NO

- Do you have diabetes mellitus (type II)?

- YES
- NO

- Do you have a diagnosis of chronic inflammatory demyelinating neuropathy (CIDP)?

- YES
- NO

- Has motor neuron disease (SLA) been suspected?

- YES
- NO

**RED FLAGS** **SECTION**

- Have you recently lost weight?

- YES
- NO

- Do you suffer from carpal tunnel on both hands?

- YES
- NO

- Do you suffer from paresthesia, tingling, numbness in your hands and/or feet? Do you lack strength in your hands and/or feet?

- YES
- NO

- Do you have a diagnosis of lumbar canal stenosis?

- YES
- NO

- Do you have balance problems (ataxia)? Have you ever fallen?

- YES
- NO

- Do you suffer from gastrointestinal disorders? (e.g. diarrhoea, constipation, early postprandial fullness, etc)

- YES
- NO

- Do you suffer from dizziness or vertigo when you move from lying or sitting to standing (orthostatic hypotension)? Do you have episodes of loss of consciousness? Do you have sexual dysfunction? (for example: erection disorder, vaginal dryness, etc...) Do you have sweating disorders? (e.g. absence of sweating)

- YES
- NO

If YES, pls. specify:…………..

- Do you suffer from cardiac problems?

- YES
- NO

- Have you ever had a bone scan?

- YES
- NO

- If YES: did it show cardiac uptake?

- YES
- NO

- Do you suffer from kidney problems?

- YES
- NO

- Do you suffer from eye problems?

- YES
- NO

- Have you ever done a biopsy confirming the presence of amyloid (TTR)?

- YES
- NO

-If YES, did it show Congo red positive deposits, compatible with amyloidosis?

- YES
- NO

- Does anyone in your family suffer from neurological problems? (e.g. difficulty walking, neuropathy, any of the symptoms discussed above, etc...)

- YES
- NO

- Does anyone in your family suffer from cardiac problems?

- YES
- NO

- Does anyone in your family have a diagnosis of hereditary transthyretin amyloidosis?

- YES
- NO
